# Predictors for pharmacological therapy and perinatal outcomes with metformin treatment in women with gestational diabetes

**DOI:** 10.3389/fendo.2023.1119134

**Published:** 2023-01-30

**Authors:** Malgorzata M. Brzozowska, Anita Puvanendran, Dana Bliuc, Andrew Zuschmann, Agata K. Piotrowicz, Anthony O’Sullivan

**Affiliations:** ^1^ The Sutherland Hospital, Endocrinology, Sydney, NSW, Australia; ^2^ UNSW Sydney, Faculty of Medicine, Sydney, NSW, Australia; ^3^ Garvan Institute of Medical Research, Healthy Ageing Theme, Sydney, NSW, Australia; ^4^ Launceston General Hospital, Endocrinology, Launceston, TAS, Australia; ^5^ Faculty of Medicine, The University of Sydney, Sydney, NSW, Australia; ^6^ St. George Hospital, Endocrinology, Sydney, NSW, Australia

**Keywords:** gestational diabetes mellitus, dietary intervention, perinatal outcomes, metformin, treatment predictors

## Abstract

**Background:**

The prevalence of gestational diabetes mellitus (GDM) has been increasing in Australia and worldwide. The study aims were to examine, in comparison with dietary intervention, perinatal outcomes for women with gestational diabetes who were attending a single hospital clinic and to identify predictors for their pharmacological GDM treatment.

**Methods:**

A prospective, observational study of women with GDM, treated with “Diet, N= 50”, “Metformin, N = 35”, “Metformin and Insulin, N = 46” or “Insulin, N = 20”.

**Findings:**

The mean BMI for the whole cohort was 25.8 ± 4.7 kg/m^2^. The Metformin group, compared to the Diet group, had OR=3.1 (95% CI:1.13 to 8.25) for caesarean section birth (LSCS) compared to normal vaginal birth mode with no longer such a significant association after controlling for the number of their elective LSCS. The insulin treated group had the highest number of small for gestational age neonates (20%, p<0.05) with neonatal hypoglycaemia (25%, p< 0.05). Fasting glucose value on oral GTT (glucose tolerance test) was the strongest predictor for a pharmacological intervention requirement with OR = 2.77 (95CI%: 1.16 to 6.61), followed by timing of OGTT with OR=0.90 (95% CI: 0.83 to 0.97) and previous pregnancy loss with OR=0.28 (95% CI:0.10 to 0.74).

**Interpretation:**

These data suggest that metformin may be a safe alternative treatment to insulin treatment in GDM. Raised fasting glucose on oral GTT was the strongest indicator that GDM women with BMI < 35 kg/m^2^ may require pharmacological therapy. Further studies are needed to identify the most effective and safe management of gestational diabetes within the public hospital setting.

**Australian New Zealand Clinical Trial Registry ANZCTR Trial Id:**

ACTRN12620000397910.

## 1. Introduction

The prevalence of gestational diabetes mellitus (GDM) has been increasing in Australia and worldwide likely due to rising average maternal age and increasing obesity especially in young adults (1). In Australia, GDM is now becoming a common complication of pregnancy as it affects around 10% of pregnancies with up to 30% of pregnancies being affected by GDM in high-risk populations (2). In most cases, GDM occurs in pregnant women with impaired pancreatic function, which is insufficient to overcome the insulin resistance associated with the pregnant state.

In recent years, GDM has been diagnosed more frequently in Australia based on more stringent diagnostic criteria recommended by the International Association of Diabetes and Pregnancy Study Groups (IADPSG), which were endorsed by the Australasian Diabetes in Pregnancy Society (ADIPS) (2). The usual time point for GDM screening is recommended to be between 24-28 weeks of pregnancy (3). Earlier screening, recommended by these expert groups, in high risk women is desirable to enable lifestyle interventions focused on diet, physical activity, and weight control to be initiated during the first or early second trimesters of pregnancy (4).

The diagnosis of GDM carries important risks of adverse short and long-term clinical outcomes for women and their offspring. The main immediate consequences of GDM are increased risks of preeclampsia, large for gestational age (LGA) newborns, and caesarean birth, with their associated perinatal co-morbidities (5). GDM is associated with up to 10-times higher odds for the development of future maternal type 2 diabetes or prediabetes in comparison with individuals with a normoglycemic pregnancy (6). Women who are affected by GDM are not only at high risk of developing type 2 diabetes later in life, furthermore, having gestational diabetes is associated with a relative risk of 2.0 (95% CI, 1.6-2.5) for being affected by future cardiovascular disease (7).

Importantly, pharmacological treatment has been shown to improve perinatal outcomes of GDM with reductions in preeclampsia, macrosomia, shoulder dystocia and neonatal death (8). Insulin has been recommended as the first-line treatment agent for GDM in the U.S (9) while in the UK, the National Institute for Health and Care Excellence (NICE) together with Scottish and Canadian guidelines recommends that metformin, an insulin sensitizer, which reduces hepatic gluconeogenesis, and increases peripheral glucose uptake (10), may be considered as initial pharmacological glucose lowering treatment in GDM women (11). Although insulin therapy has been shown to reduce the risk of neonatal macrosomia and rate of serious perinatal outcomes such us shoulder dystocia or perinatal death (12), the benefits of insulin treatment in pregnancy often do not extend to preventing neonatal hypoglycaemia, frequently requiring intravenous glucose infusion and neonatal high-level nursery admission (13). Furthermore, gestational insulin therapy requires additional education, resources and training with the need for increased care for women throughout pregnancy and the act of injecting insulin can be stressful for some women.

At our institution, in our cohort of pregnant women with GDM, metformin has been endorsed as an alternative treatment to insulin therapy. We have therefore hypothesized that metformin use to treat GDM will result in similar pregnancy outcomes in comparison to pregnant women who are treated with insulin alone.

The present study examined perinatal outcomes for women with GDM who were treated with pharmacological interventions in comparison with dietary lifestyle changes alone while controlling for differences in baseline maternal characteristics. In particular, the primary aim was to examine the differences in composite maternal and in neonatal outcomes between four GDM treatment groups (“Diet”, “Metformin”, “Metformin and Insulin”, “Insulin”). In addition, specific maternal and neonatal outcomes were also reported and analysed separately. The second aim of the study was to identify early clinical maternal predictors for the use of pharmacological treatment in pregnancy affected by GDM.

## 2. Materials and Methods

### 2.1. Study design

We have conducted a prospective, observational, cohort study through a review of the medical records of women with GDM in singleton pregnancy who attended the multi-disciplinary Gestational Diabetes Clinic at Sutherland Hospital, Sydney, Australia, between years 2016 to 2018. The analyzed data were consecutively extracted from electronic and from hard copies of medical records.

Weight was measured to the nearest 0.1 kg on a digital scale (TANITA, Wedderburg) and height was measured to the nearest 0.1 cm with a scale-mounted stadiometer during the first antenatal visit. BMI (kg/m^2^) was calculated. Women with Type 1 and 2 pre-gestational diabetes as well as women with BMI exceeding 35 kg/m2 were excluded from the analysis, as their care was transferred to the tertiary referral centre. Furthermore, in our institution women who underwent previous lower segment caesarean sections (LSCS) were not being offered an option of vaginal birth after caesarean delivery (VBAC).

The Southern Eastern Sydney Local Health District Human Research Ethics Committee (Study Reference No. RESP/15/107) approved the study. The study was registered with Australian New Zealand Clinical Trial Registry ANZCTR Trial Id: ACTRN12620000397910. This cohort study, in accordance with the current rules of the local Research Ethics Committee, did not require the patient’s informed consent.

### 2.2. GDM diagnosis and treatment

GDM was diagnosed, as recommended for Australian women who are at 24–28 weeks gestation, using a 75-g oral glucose tolerance test (OGTT) following an overnight fast, applying the new diagnostic criteria, introduced in 2015, of a fasting plasma glucose ≥ 5.1 mmol/L, a 1-hour plasma glucose ≥ 10 mmol/L or a 2-h plasma glucose ≥ 8.5 mmol/L, as endorsed by ADIPS (14, 15). In our institution early screening (i.e., before 24 weeks gestation) is performed in high-risk patients including those with previous GDM, or other risk factors for GDM (pre-pregnancy BMI > 30 (kg/m2), previous birth of baby with birthweight above 4000 grams, family history of diabetes or those of a high-risk ethnicity)) (16).

All women diagnosed with GDM attended two separated education sessions containing dietary and lifestyle advice in pregnancy, which were run by a Diabetes Nurse Educator and Dietician. Women were advised to monitor their blood glucose levels (BGLs) 4 times daily using a blood glucose meter: in a fasting condition as well as at 2-hours post breakfast, lunch and dinner. Women were advised to follow a carbohydrate modified diet (30–45 grams of carbohydrate at main meals, 15–30 grams of carbohydrates at mid meals) and they were encouraged to consume low glycaemic index carbohydrates. In our institution, following Endocrinology advice, GDM women would commence on the pharmacological management when their BGLs, despite lifestyle and dietary modification, were exceeding fasting BGLs =< 5.0 and = <6.7 mmol/L 2-h postprandially. In line with NICE guidelines metformin use was discussed as first line of pharmaceutical therapy (11) together with an alternative choice of insulin treatment as dependent upon patient and physician preference. Insulin alone was a preferred treatment in high-risk women who due to their multiple risk factors underwent earlier OGTT. The insulin was commenced (insulin isophane and/or insulin aspart) based on the pattern of hyperglycaemia. Insulin doses were titrated to target fasting and postprandial BGLs by the treating Endocrinologist. The metformin group included GDM women who were prescribed metformin as the first line therapy. The initial metformin dose was 500 mg daily, which was up-titrated to 2000 mg per day (where tolerated) to aim for adequate glycaemic control. Treatment was intensified by the addition of insulin in women who did not achieve adequate glycaemic control with metformin alone.

Therefore, study patients were prospectively allocated to one of four treatment exposure groups (“Diet”, “Metformin”, “Metformin and Insulin”, “Insulin”).

#### 2.2.1 Main primary outcome measure

The main composite study aim was to examine the differences in maternal and in neonatal outcomes between four GDM treatment groups. These perinatal outcomes were: maternal outcomes—mode and gestational age at delivery, timing of delivery and neonatal outcomes— neonatal birth weight, preterm birth, indicated by spontaneous birth before 37 weeks’ gestation; large-for-gestational-age (LGA; defined as birth weight > 90th centile for gestational age and gender), small-for-gestational-age (SGA; defined as birth weight (SGA; defined as birth weight < 10th centile for gestational age and gender) (17), presence of shoulder dystocia, neonatal respiratory distress, neonatal hypoglycaemia, jaundice, birth injury and neonatal death. The composite outcome was a binary variable defined as 1 if at least one maternal or neonatal outcome was present, or 0 in the absence of both maternal and neonatal outcomes.

#### 2.2.2 Secondary outcomes measure

In order to identify the secondary study aim three pharmacological interventions were grouped together. For this aim, participants were classified in two groups: 1) any pharmacological intervention, including “Metformin”, “Metformin and Insulin”, and “Insulin” groups and 2) “Diet”.

#### 2.2.3 Neonatal hypoglycaemia

In our institution the presence of formal BGL < 2.6 mmol/L in neonates who are less than 48 hours of age warrants immediate intervention. These neonates are admitted to the neonatal intensive care unit (NICU) for treatment. The definition of neonatal hypoglycaemia is based on the study, which demonstrated reversible disturbance in evoked potentials at BGL < 2.6mmol/l in a small cohort of asymptomatic term babies (18).

The aim of hypoglycaemia treatment is to return the neonatal BGL values to their safe range (> 3.9 mmol/L) through normal nutritional intake. For BGLs ranging from 1.5 to 2.5 mmol/L this occurs through the use of oral 40% Dextrose Gel, which is massaged into neonatal buccal mucosa, followed by refeed with either breast or formula. Severe symptomatic hypoglycaemia is corrected with an IV 10% dextrose bolus at 2 mL/kg and infusion at 60 – 80 ml/kg/day or IM glucagon. High risk neonates are monitored for at least the first 24 hours of life in NICU until the neonate’s BGLs remain at safe levels (≥ 2.6mmol/L) for at least 24 hours after the last episode of hypoglycaemia.

### 2.3. Statistical analysis

Baseline study data are presented as mean ( ± SD) for normally distributed variables and median (interquartile range) for non-normally distributed variables. Analysis of variance (ANOVA) and *post-hoc* pairwise Tukey honest significance difference test, or Kruskal–Wallis test and *post-hoc* pairwise Dunn’s test were used to examine the imbalance between the study groups for normally or non-normally distributed baseline data, respectively. Categorical variables are presented as number (%), and Fisher’s exact test was used for the between group comparisons.

The association between pharmacological intervention for GDM and adverse perinatal outcomes was determined using unadjusted and multivariable adjusted logistic regression analyses.

An exploratory analysis examined an association between mode of birth and treatment procedures after adjustment for the differences in baseline characteristics between study groups such as fasting glucose and BMI.

The secondary outcome of the study was to identify early clinical maternal predictors for the pharmacological treatment in GDM. The analyzed study data included maternal characteristic defined as age, ethnicity, body mass index (BMI), family history of diabetes, parity, pooled number of previous miscarriages and pregnancy terminations, previous history of GDM, history of thyroid disease and thyroid stimulating hormone (TSH) values, vitamin B12 levels, 25 (OH) D levels and the timing of OGTT with BGL values on OGTT and gestational age at diagnosis of GDM. The fasting glucose was analyzed as a continuous variable to avoid misclassification error of exposure variable.

Variables were firstly screened in univariate analysis, and those with a p-value < 0.25 were included in the multivariable model. The final model was selected using stepwise regression.

Statistical analyses were conducted using R software version 4.0.4 (2021–02–15) with P value of < 0.05, which was considered statistically significant.

## 3. Study results

### 3.1. Baseline characteristics of study patients

During the time period of September 2016 to April 2018, 151 women were identified as being treated with Diet (N = 50), Metformin (N = 35), taking Metformin and Insulin (N=46) or with Insulin alone (N=20) during singleton GDM pregnancy. The demographics of these groups are outlined in **Table 1**. There were differences in baseline characteristics between study groups in subjects’ height, weight, timing of their OGTT, value of fasting BGL on OGTT, family history of diabetes and total vitamin B12 (**Table 1**).

**Table 1:** Baseline characteristics of study subjects.

	All	Diet	Metformin	Metformin And Insulin	Insulin	P -value
Number	151	50	35	46	20	
Ethnicity						
Caucasian	90 (60%)	32 (64%)	19 (54%)	29 (63%)	10 (50%)	0.23
South Asian	27 (18%)	5 (10%)	4 (11%)	10 (22%)	8 (40%)	
East Asian	17 (11%)	6 (12%)	5 (14%)	5 (11%)	1 (5%)	
South-East Asian	13 (9%)	5 (10%)	6 (17%)	2 (4%)	0 (0%)	
Middle Eastern	3 (2%)	1(2%)	1 (3%)	0	1 (5%)	
Height (cm)	162 ± 7.3	164 ± 6.2	159 ±. 6.2	163 ± 7.1	158 ± 9.4**	**0.006**
Weight (kg)	67.4 ± 13.5	64.6 ± 13.3	62.5 ± 9.9	72.2± 13.6*	69.4 ± 15.7	**0.009**
BMI (kg/m^2^)	25.8 ± 4.7	24.6 ± 4.7*	24.6 ± 3.9	27.0 ± 4.3*	27.6 ± 5.6**	**0.009**
Age (years)	31.9 ± 4.9	31.4 ± 4.6	31.5 ± 5.0	32.3 ± 4.9	32.9 ± 5.8	0.61
Parity	0.8 ± 1.1	0.8 ± 1.0	0.5 ± 0.6	0.9 ± 0.9	1.3 ± 1.9	0.10
Gravidity	2.4 ± 1.6	2.4 ± 1.5	1.9 ± 1.2	2.4 ± 1.4	3.0 ± 2.3	0.12
Family History of DM	38/121 (31%)	16/42 (38%)	6/25 (24%)	6/36 (17%) *	10/18 (56%)	**0.019**
Miscarriages (N, %)	49 (33%)	19 (38%)	9 (26%)	15 (33%)	6 (30%)	0.094
History of thyroid disease	28 (19%)	8 (16%)	4 (11%)	11 (24%)	6 (30%)	0.29
TSH (mIU/L)	1.6 ± 0.88	1.5 ± 0.78	1.4 ± 0.94	1.7 ± 0.82	2.1± 1.18**	0.16
Active Vitamin B12 (pmol/L)	78 ± 36	78 ± 25	73 ± 24	91 ± 57	52 (47, 58)	0.49
Vitamin B12 (pmol/L)	264 ± 96	286 ± 99	328 ± 119^	248 ± 70	182 ± 8	**0.028**
25 (OH) D (pmol/L)	94 ± 31	96 ± 29	87 ± 36	95 ± 26	82 ± 60	0.75
Past GDM (N, %)	45 (30%)	14 (28%)	7 (20%)	17 (37%)	7 (35%)	0.49
OGTT (weeks)#	27 (17.2, 28)	28 (27, 29)	27 (20, 29)	21 (15.2, 28) *	21 (13.5, 27.5) **	**0.006**
FBGL (mmol/L) on OGTT	4.8 ± 0.6	4.6 ± 0.5	4.7 ± 0.6	5.0 ± 0.5*	5.2 ± 0.5**	**0.0001**
(+ 60 min) BGL	9.6 ± 2.0	9.9 ± 1.7	9.6 ± 1.6	9.1 ± 2.3	10 ± 2.5	0.25
(+120 min) BGL	7.8 ± 1.7	7.9 ± 1.7	8.0 ± 1.3	7.5 ± 1.7	8.2 ± 2.6	0.45
Metformin dose (mg)	971 ± 356		924 ± 383	1006 ± 334		0.32
Metformin start (weeks)	28 ± 6.3		31 ± 4.3	26 ± 6.8		**0.001**
Metformin duration (weeks)	11 ± 6.1		8 ± 4.3	12 ± 7.8		**0.011**
Insulin start (week)	26 ± 7.4			27 ± 6.6	23 ± 8.3	0.072
Insulin dose (units)#	20 (10, 31)			14.5 (9, 28)	31 (13, 39)	0.48
Initial HbA1c (%) (mmol/mol)	5.2 ± 0.4 (33)	5.2 ± 0.4 (33)	5.2 ± 0.3 (33)	5.1 ± 0.3 (32)	5.4 ± 0.3 (36)	0.45
Antenatal US (weeks)	35 ± 1.8	35 ± 1.7	35 ± 1.8	34 ± 2.0	35 ± 1.7	0.32
Abdominal Circumference (%) #	60 (40, 85)	68 (50, 85)	50 (45, 90)	53 (35, 77)	44(14, 81)	0.35
EFW (%) #	50 (42, 75)	50 (43, 71)	61 (50, 85)	56 (40, 70)	46 (16, 66)	0.22
Hypertension	12 (8%)	6 (12%)	2 (6%)	3 (7%)	1 (5%)	0.58
Abnormal Liquor	3 (2%)	1 (2%)	0	1 (2%)	1 (5%)	0.60
Proteinuria	4 (3%)	1 (2%)	1 (3%)	0	2 (10%)	0.12
Steroid use	13 (9%)	3 (6%)	1(3%)	7 (15%)	2 (10%)	0.25

^Metformin versus Diet, * Metformin and Insulin versus Diet **Insulin versus Diet.

Values are expressed as median and interquartile range (IQR). FBGL, fasting blood glucose level; BGL, blood glucose level; OGTT, oral glucose tolerance test; BMI, body mass index.Bolded values indicate statistical significance.

Compared to Diet, Metformin and Insulin and Insulin groups were heavier (p = 0.009). In addition, Metformin group, although not on vitamin B12 supplementation, had higher level of total vitamin B12 (p = 0.028**),** Metformin and Insulin group were more likely to have family history for diabetes (p = 0.019). There were no differences in age, the number of previous pregnancies and live births, number of previous pregnancies affected by GDM, initial HbA1c level, previous thyroid disease, TSH or 25 (OH) vitamin D levels.

### 3.2. Treatment of gestational diabetes

Metformin and Insulin and Insulin alone groups, as having identified risk factors for the GDM at their first antenatal visit, had earlier OGTTs, an average at 21 weeks, in comparison with the Diet treated group which had an average OGTT at 28 weeks (p = 0.006). There was no difference in the history GDM in previous pregnancies between study groups or in their HbA1c with an average initial HbA1c of 5.2% (± 0.34), (33 mmol/mol). Approximately a third of these pregnant women were diagnosed with GDM in their previous pregnancies and experienced previous spontaneous miscarriages or terminations of their pregnancies.

Women that were treated with insulin or with metformin and insulin had significantly higher fasting glucose on 75 g OGTT (5.19 mmol/L or 5.01 vs. 4.6 mmol/L, respectively, p= 0.0001), in comparison with women treated with diet and lifestyle modification alone without such difference for their 1- hourly and 2- hourly BSL. Caucasian women had a higher mean fasting BGL of 4.9 mmol/L (SD = 0.55), p = 0.015 on oral GTT in comparison with a mean fasting BGL of 4.7 mmol/L (SD = 0.56) in women of Asian ethnicity.

The timing of pharmacological intervention varied between groups. Women treated with insulin were initiated on their therapy earlier at 23 ± 8.3 weeks while women treated with metformin only on average commenced on metformin at 31 ± 4.3 weeks. The mean gestational age at which insulin was added to metformin was 27 ± 6.6 weeks (**Table 1**).

There was no difference in the total daily dose of insulin at delivery for women in the Metformin and Insulin and Insulin alone groups. There was no difference in the foetal abdominal circumference on the antenatal scans (34-36 weeks).

### 3.3. Perinatal (maternal and neonatal) outcomes

Maternal and neonatal outcomes for women taking metformin (with or without additional insulin) in comparison with those managed with diet and lifestyle modification are outlined in **Table 2**. There was no overall difference in maternal and neonatal composite study outcomes between groups (p = 0.13), (**Table 2**). There was no difference in ethnicity distribution between groups (**Table 1**). Furthermore, there was no interaction between study procedures and the ethnicity of women with GDM in primary composite study outcome (p = 0.38). In particular, there was no overall difference between study groups in the rate of normal vaginal birth (NVB) (p = 0.19), instrumental vaginal birth (p = 0.14) or LSCS (p = 0.36) or in the gestational age of the time of birth (p = 0.17). However, the comparison of each treatment group to the dietary intervention revealed that metformin treated group had 3.1 times the odds (95% CI: 1.13 to 8.25) for the birth by LSCS with the trend for the positive association between metformin treatment and instrumental vaginal birth (**Table 3**). Furthermore, once the effect of the treatment procedure on mode of birth was adjusted for the value of fasting glucose on subjects’ OGTT and their initial BMI this association became stronger with OR = 3.4 (95% CI: 1.04 to 8.86) for birth by LSCS for the metformin treated group and with OR = 11.12 (1.18 to 104.71) for the instrumental vaginal birth (**Table 3**). Once we excluded elective LSCS from the analysis, Metformin and Insulin group had lower rate of LSCS than Metformin group without difference to the Diet treated group (**Table 2**).

**Table 2:** Maternal and neonatal outcomes of study patients.

	Diet N=50	Metformin N=35	Metformin & Insulin N=46	Insulin N=20	Metformin vs Diet OR (95%CI)	Metformin & Insulin vs Diet OR (95%CI)	Insulin vs Diet OR (95%CI)
**Primary Composite outcome (N, %)**	27 (54)	26 (74)	26 (57)	16 (80)	2.3 (0.89 to 5.82)	1.2 (0.54 to 2.76)	3.1 (0.92 to 10.75)
Maternal Outcomes							
Mode of Birth							
Vaginal, (N, %)	35 (70)	15 (43)	27 (59	12 (60)	**0.4 (0.15 to 0.94)**	0.6 (0.26 to 1.52)	0.6 (0.19 to 1.67)
Instrumental, (N, %)	5 (10)	6 (17)	5 (11)	3 (15)	2.7 (0.60 to 12.16)	1.1 (0.21 to 5.71)	2.8 (0.51 to 15.04)
LSCS, (N, %)	10 (20)	**14 (40) ^**	14 (30)	5 (25)	**2.8 (1.06 to 7.41)**	1.8 (0.69 to 4.46)	1.3 (0.39 to 4.55)
Emergency LSCS, (N, %)	7 (70)	**10 (71) °**	4 (29)	4 (80)	2.9 (0.95 to 9.03)	0.5 (0.12 to 2.18)	1.8 (0.46 to 7.35)
Neonatal outcomes							
Gestational age at birth (weeks)	39 ± 1.3	39 ± 0.8	39 ± 1.4	39 ± 0.9			
Birth weight (gr)	3360 (443)	3228 (507)	3194 (452)	3124 (684)			
LGA, (N, %)	2 (4)	2 (6)	2 (4%)	2 (10)	1.5 (0.19 to 10.85)	1.1 (0.15 to 8.27)	2.8 (0.37 to 21.64)
SMA, (N, %)	2 (4)	4 (11)	3 (7)	4 (2) **	2.0 (0.42 to 9.66)	2.4 (0.55 to 10.0)	**5.6 (1.19 to 26.38)**
Premature birth, (N, %)	2 (4)	2 (6)	5 (11)	1 (5)	3.0 (0.26 to 34.48)	6.0 (0.67 to 53.49)	
Shoulder dystocia, (N, %)	2 (4)	2 (6)	3 (6)	2 (10)	1.5 (0.20 to 11.29)	1.7 (0.27 to 10.67)	1.3 (0.11 to 15.37)
Respiratory distress, (N, %)	4 (8)	2 (6)	2 (4)	2 (10)	0.7 (0.13 to 4.2)	0.8 (0.17 to 3.86)	1.3 (0.22 to 7.92)
Hypoglycemia, (N, %)	6 (12)	3 (9)	6 (13)	**5 (25) ^#^**	0.5 (0.12 to 1.85)	0.96 (0.29 to 2.52)	2.8 (0.86 to 9.04)
Post-partum haemorrhage, (N, %)	7 (14)	5 (14)	6 (13)	3 (15)	1.2 (0.44 to 3.31)	0.3 (0.09 to 1.04)	0.8 (0.23 to 2.96)
Neonatal complications	Bilateral pyelectasis (N=1), cleft palate (N=1)		Positional talipes (N = 1)	Hypospadia- sis (N=1)			

Number (N) of participants with events and Odds Ratios with 95% Confidence intervals for the primary composite event and specific maternal and neonatal outcomes. Sample size was too small to calculate OR for Premature Birth for comparison between Insulin to Diet groups.

LGA- large for gestational age, SGA – small for gestational age.

Bolded values indicate statistical significance. ^ Metformin versus Diet, ** Insulin versus Diet, ° Metformin versus Metformin and Insulin, # P-Values are for the differences between treatment groups.

**Table 3:** Effect of GDM treatment procedure on mode of birth.

	LSCS vs NVB		ID VS NVB	
Study treatment groups	**OR (95% CI)**	**P - value**	**OR (95% CI)**	**P - value**
Metformin vs diet	3.06 (1.13 to 8.25)	**0.03**	4.00 (0.85 to 18.90)	0.08
Metformin and insulin vs diet	1.58 (0.62 to 4.00)	0.33	1.24 (0.23 to 6.62)	0.80
Insulin vs diet	1.36 (0.39 to 4.73)	0.62	3.00 (0.53 to 16.19)	0.21
Effect of treatment on mode of birth controlled for fasting BGL on subjects’ GTT and BMI				
	**LSCS vs NVB**		**ID VS NVB**	
Study treatment groups	**OR (95% CI)**	**P - value**	**OR (95% CI)**	**P - value**
Metformin vs diet	3.04 (1.04 to 8.86)	**0.04**	11.12 (1.18 to 104.71)	**0.04**
Metformin and insulin vs diet	1.66 (0.59 to 4.70)	0.34	1.03 (0.06 to 18.11)	0.98
Insulin vs diet	2.22 (0.54 to 9.09)	0.27	10.32 (0.85 to 124.66)	0.07
Fasting BGL (GTT)	1.04 (0.48 to 2.25)	0.92	1.32 (0.38 to 4.51)	0.66
BMI	0.99 (0.91 to 1.08)	0.83	1.00 (0.85 to 1.17)	0.96

LSCS, Caesarean section; NVB, normal vaginal birth; ID, instrumental vaginal birth; BGL, blood glucose level; BMI, body mass index; OR, Odds Ratios. Bolded values indicate statistical significance.

There were no differences between subjects treated with pharmacological intervention and dietary/lifestyle modifications in birth weight, numbers of shoulder dystocia, cases of respiratory distress, postpartum haemorrhage, rates of premature delivery or large-for-gestational-age neonates (**Table 2**). There were 19 neonates who required NICU admission: Diet with N = 7 (14%), Metformin with N=3 (9%), Metformin and Insulin with N = 7 (15%), Insulin with N = 2 (10%). The 8 newborns were separated from their mothers due to admission to NICU caused by: intrauterine growth retardation due to oligohydramnios (2 infants), significant hypoglycaemia (2 infants), respiratory distress requiring CPAP due to meconium aspiration (2 infants), foetal bradycardia (1 infant) and feeding problems due to undiagnosed cleft palate (1 infant).

One- fifth of insulin treated women delivered children who were small for their gestational age. The neonates in insulin alone treated group had the highest proportion (25%) of hypoglycaemic episodes however two of these children were affected by prematurity and by intrauterine growth retardation, respectively. The mothers of neonates with hypoglycaemia, prior to delivery, had high daily insulin requirements, which ranged between 58 units to 104 units.

### 3.4. Changes in vitamin B12 over time

The were 78 women who had measured total vitamin B12 level at their initial visit. The 8 of them (10%) were noted to have vitamin B12 level below the reference range (RR 150-700 pmol/L). When the cohort of these women was analyzed together, at their baseline and approximately 8 weeks later, there was a reduction in measured total vitamin B12 level from baseline vitamin B12 of 264 ± 96 pmol/L (RR 150-700pmol/L) to vitamin B12 of 242 ± 71 pmol/L (p = 0.019). Due to the limitation of small sample size, we were unable to compare differences between intervention groups.

### 3.5. Maternal predictors for the pharmacological treatment in pregnancy

#### 3.5.1 Univariate analysis

In the univariate analysis the following variables were positively associated with the need for pharmacological GDM treatment: BMI with OR = 1.1 (95% CI; 1.00 to 1.18, p=0.038), timing of the OGTT in pregnancy with OR = 0.9 (95%CI; 0.86 to 0.98, p=0.0059), fasting BGL values on OGTT with OR = 3.1 (95% CI: 1.50 to 6.47, p=0.0024) and inversely with number of previous miscarriages with OR = 0.5 (95% CI: 0.23 to 1.07, p= 0.075).

There was no association between need for pharmacological GDM treatment and following variables: ethnicity (p = 0.10), age (p = 0.47), history of previous GDM (p = 0.83), TSH level (p = 0.32), previous thyroid disease (p = 0.61), 1-hourly BSL on OGTT (p = 0.20), 2-hourly BSL on OGTT (p = 0.20), initial vitamin B12 level (p = 0.82), initial 25 (OH) D level (p = 0.40) and with positive family history for DM (p = 0.25).

#### 3.5.2 Logistic Regression (Multivariate Analysis).

In the multivariate analysis, the timing of OGTT and personal history of miscarriages or terminations of pregnancy were inversely and significantly associated with need for pharmacological treatment while fasting BGL was a strong and positive predictor of the need for escalating treatment intervention. Maternal BMI value was no longer significantly associated with the need for pharmacological therapy (**Table 4**).

**Table 4:** Univariate and Multivariate analysis with predictors of pharmacological therapy in patients with gestational diabetes.

Univariate analysis			
	Odds Ratio	(95% CI)	P - value
BMI (kg/m^2^)	1.1	(1.00 to 1.18)	**0.038**
OGTT (weeks)	0.9	(0.86 to 0.98)	**0.0059**
FBGL (mmol/L) (OGTT)	3.1	(1.50 to 6.47)	**0.0024**
Personal history of past miscarriage	0.5	(0.23 to 1.07)	0.075
Ethnicity (Caucasian)	0.54	(0.27 to 1.10)	0.09
Age (years)	1.02	(0.96 to 1.10)	0.47
Past GDM	1.11	(0.49 to 2.54)	0.83
(+ 60 min) BGL (mmol/L)	1.14	(0.94 to 1.38)	0.20
(+ 120 min) BGL (mmol/L)	1.03	(0.84 to 1.28)	0.76
TSH level (mIU/L)	0.78	(0.47 to 1.28)	0.32
Personal history of thyroid disease	1.21	(0.46 to 3.18)	0.61
25 (OH) D level (pmol/L)	0.90	(0.98 to 1.01)	0.40
Vitamin B12 level (pmol/L)	1.0	(1.0 to 1.01)	0.81
Family history of DM	1.59	(0.72 to 3.53)	0.25
Multivariate Analysis			
	**Odds Ratio**	**(95% CI)**	**P - value**
Fasting BGL (mmol/L) (OGTT)	2.77	(1.16 to 6.61)	**0.022**
Oral GTT (weeks)	0.90	(0.83 to 0.97)	**0.008**
BMI (kg/m^2^)	1.05	(0.95 to 1.17)	0.38
Previous miscarriage	0.28	(0.10 to 0.74)	**0.010**

FBGL, fasting blood glucose level; OGTT, oral glucose tolerance test; BMI, body mass index; DM, diabetes mellitus.Bolded values indicate statistical significance.

## 4. Discussion

The results from the present study support our hypothesis that metformin treatment (alone or combined with insulin) of women with GDM does not result in worse pregnancy outcomes as compared with those who were assigned to insulin. Despite no significant difference in main composite study aim between study groups and Diet group, insulin treatment alone, although prescribed for small number of GDM patients, was associated with a higher proportion of “small for gestational age” neonates and higher rates of neonatal hypoglycaemia in comparison with dietary or metformin treated groups. Such neonatal complications likely resulted from worse glycaemic control in this group of patients as indicated by their high daily maternal insulin requirements, which ranged between 58 units to 104 units.

Importantly, previous large retrospective cohort study reported that pregnancy outcomes are worse in GDM women with SGA neonates than in those without GDM with subsequent increased risks for respiratory distress syndrome, intrauterine foetal death, hypoglycaemia, jaundice and neonatal demise (19).Interestingly however, the metformin treated group in comparison with dietary intervention group had a higher risk for LSCS. Such association between mode of birth and metformin treatment is related not only to the treatment but also to the differences in our subjects basal characteristic. Previous studies have highlighted that, increased maternal BMI either in overweight or obese category without GDM, increased the risk of macrosomia and caesarean delivery when compared to normal weight women (20). In our metformin treated cohort we observed no evidence of foetal growth acceleration on third trimester ultrasound in majority of patients, likely reflective of satisfactory maternal glucose control (21). Indeed, once we excluded elective LSCS from the analysis there was no difference in number of emergency LSCS in metformin group in comparison with dietary intervention.

To our knowledge no randomised trials (RCTs) compared effects of metformin directly to dietary/lifestyle intervention in pregnancy, although several previous studies compared metformin and insulin interventions alongside dietary interventions for both trial arms. The analysis of 16 RCTs or follow-up of a RCTs revealed that metformin in comparison with insulin treatment did not increase the risk of caesarean section (RR = 0.97; 95% CI, 0.80 to 1.19) (22). Furthermore, the meta-analysis consisting of 11 trials reported that women randomised to metformin had lower risk for adverse maternal and neonatal outcomes including lower risk for the instrumental delivery compared to those randomised to insulin (23). The above data points to metformin being a useful alternative to insulin therapy with a high degree of patient acceptability.

Importantly, in comparison with insulin, metformin can significantly decrease maternal weight gain, and therefore metformin is now the preferred treatment option for an increasing number of women with a BMI in obese category (24). The efficacy of metformin treatment in GDM is not without limitation, as congruent with present study, approximately 14% to 46% of pregnant women fail to achieve adequate glycaemic control with metformin alone (25).

The lack of longer-term safety studies and that metformin can cross the placenta raise potential concerns associated with metformin therapy in pregnancy. The safety and optimal metformin doses in pregnancy have not been yet defined, however most studies use metformin doses ranging from 500 mg to 2500 mg a day (22). In the present study the metformin treatment was commenced in later stages of pregnancy, on average at 28 ± 6.3 weeks of pregnancy, therefore without effect on early embryonic growth.

To date, no increased risk for non-genetic congenital anomalies has been identified following foetal exposure to metformin during the first trimester of pregnancy. A randomized, placebo-controlled trial of PCOS women who were either randomised to metformin (500 mg twice daily increasing to 1000 mg twice daily) or placebo from the first trimester gestational age between 5 and 12 weeks found no difference in the primary composite study outcome of preeclampsia, GDM and preterm delivery (26). Furthermore, no adverse safety signal was detected in randomised, placebo controlled trial of pregnant women with type 2 diabetes in pregnancy who were randomised either to metformin or placebo at 16.5 weeks of pregnancy. This study found no significant difference in congenital anomaly with 7/227 (3%) affected infants in metformin treated group in in comparison with 13/227 (6%) infants in dietary intervention group (p value of 0.16, RR 0.52 (0.22 to 1.28)) (27). Reassuringly, in previous studies, exposure *in utero* in children of GDM women to metformin ( ± insulin) or insulin alone led to similar total and abdominal body fat percent and metabolic measures at children at 7–9 years of life (28). Conversely, in a recent study, metformin exposure in the first trimester of pre-gestational diabetes was associated with an increased risk of birth defects and pregnancy loss; however, these adverse pregnancy outcomes were attributed to underlying disease rather than to metformin therapy (29). On-going long term follow-up studies of children born to mothers affected by GDM will help answer this current uncertainty.

In the present study, only 33% of GDM patients achieved satisfactory glycaemic control through the dietary therapy while the majority of pregnant GDM women (77%) required pharmacological treatment. The ability to predict a-priori which GDM group of patients will fail their dietary intervention would help to plan steps more effectively in their GDM management.

Past studies have identified the following predictors of the requirement to introduce pharmacological treatment in GDM. These predictors included early GDM diagnosis (e.g., at <25 weeks gestation), a family history of diabetes, non-European ethnicity, an older age, elevated fasting blood glucose level, HbA1c at GDM diagnosis, and an elevated pre- pre-pregnancy BMI (20, 30). In our unique group of pregnant non-obese women, we have identified fewer predictors of the need for pharmacological GDM treatment. Those predictors included maternal characteristics such as baseline maternal BMI, value of fasting BGL on their OGTT, the number of previous miscarriages and early GDM diagnosis (e.g., at <24 weeks gestation). Although on average Caucasian women had a higher fasting glucose on their GTT than women of Asian ethnicity, their ethnicity was not a significant predictor of the need for escalating GDM therapy beyond diet alone. Maternal BMI, once controlled for the fasting BGL level on OGTT and timing of the OGTT and number of previous miscarriages, was no longer a significant indicator of the need for pharmacological GDM therapy.

In previous research, elevated pre-pregnancy maternal BMI predicted failure of dietary therapy (20) with higher maternal BMI being significantly associated with the need for medical treatment (31–33). Although obesity is associated with increasing insulin resistance and pancreatic β-cell dysfunction, it remains unclear whether weight control during pregnancy, as recommended by the Institute of Medicine, would reduce the risk of GDM or the need for insulin therapy (34). Considering that our study patients had an average BMI close to the normal range at 25.8 ± 4.7 kg/m^2^, we may hypothesise that having an initial BMI in the obese category would have been more closely associated with the need for pharmacological GDM intervention.

In the present research, the value of fasting glucose level on the OGTT was the strongest indicator that women with GDM may not respond to the dietary intervention alone. This study finding is important considering that some at risk women are unable to complete OGTT in pregnancy. Interestingly we have also found the increased risk for LSCS and instrumental vaginal birth with raised fasting BGL in metformin treated group. Interestingly, once the effect of metformin treatment on mode of birth was controlled for fasting glucose on subjects’ OGTT and subjects initial BMI, the risk for LSCS or instrumental vaginal birth increased significantly in metformin treated GDM women. The previous retrospective cohort study of 14,741 pregnant women found that fasting hyperglycaemia was associated with increased risk for caesarean birth (OR: 1.33, 95% CI: 1.15-1.55,P < 0.001) (35). Such strong positive association between fasting hyperglycaemia on OGTT and adverse perinatal outcomes, including caesarean birth, was noted in previous systematic review and meta-analysis of GDM women (36).

Multiple studies have highlighted the link between fasting hyperglycaemia in the first and 2^nd^ trimester of GDM pregnancies with increased occurrence of adverse pregnancy outcomes including the need for surgical birth (4, 37, 38). In previous study only fasting plasma glucose value on the oral glucose tolerance test in pregnancy was significantly associated with pregnancy adverse outcomes, irrespectively of pharmacological intervention (39). Therefore, effective treatment of fasting BGL in women affected by GDM may potentially improve maternal and neonatal health outcomes with a great potential for the early detection of women at risk of having more adverse perinatal outcomes, irrespective of their other risk factors, such as obesity and maternal age.

Interestingly, we observed that having at least one previous miscarriage or pregnancy termination may influence the need for pharmacological treatment for GDM women. Several murine studies have reported that progesterone, which is essential to sustain pregnancy, promotes insulin resistance by multiple mechanisms during pregnancy (40–42). Interestingly, previous case control study of 1567 Korean women demonstrated that threatened miscarriage is associated with decreased risk of GDM and the severity of glucose intolerance (43). Conversely, a retrospective cohort study found that having a spontaneous miscarriage was linked to a higher risk for having subsequent gestational diabetes (44). Further research is recommended to confirm these relationships and to evaluate the pathophysiologic mechanisms that interplay between these common obstetric complications.

Several studies have shown that vitamin B12 status during pregnancy is important to the health of mother and her offspring (45). In the present study, limited by the small sample size, approximately 10% of women had total vitamin B12 level in the insufficiency range and their vitamin B12 levels declined significantly during pregnancy. As metformin treatment may reduce ileal absorption of dietary vitamin B12 (46), GDM women treated with metformin would likely benefit from monitoring of their vitamin B12 status.

Our study is not free of limitations, due to pragmatic method of data collection as part of subjects’ routine clinical care, rather than at fixed short time intervals. Therefore detailed trajectories of weight gain and glycaemic control during pregnancies were not analysed in this study. However an absence of foetal growth acceleration on third trimester ultrasound in the majority of patients was likely reflective of their satisfactory maternal glucose control (21). Exclusion from the analysis of severely obese women with BMI> 35 kg/m^2^ might have reduced the risk of their pregnancy complications and a rate of instrumental or caesarean birth. Due to our small number of adverse perinatal events, and in order to improve the ability to detect differences in the primary study endpoints as well as to increase study statistical power, we have designed the main composite study outcome of combined maternal and neonatal events. Additionally, we have reported detailed information on clinically important events of which the composite outcome is based on, with their measures of association (**Table 2**). Additional advantage of this cohort study is consistent and uniform nutritional counselling as well as consistency of care being provided by the same physician.

Our clinical cohort study was relatively small. It is possible that with a bigger cohort, the association between pharmacological interventions and adverse perinatal outcomes may reach statistical significance. However, for the second study aim, when we grouped all pharmacological interventions together, we were able to identify significant predictors for the need of pharmacological treatment in GDM.

In summary, our study addressed the paucity of existing data comparing the effect of metformin intervention in pregnancy with dietary/lifestyle intervention in women with BMI below 35 kg/m^2^ and gestational diabetes. The present study has highlighted that metformin treatment of GDM may not be associated with different pregnancy outcomes compared to the GDM managed by diet except for the increased risk for the LSCS. These study findings, however, were no longer significant once the analysis was controlled for the number of elective caesarean sections.

We have also observed that elevated fasting blood glucose on OGTT, in metformin treated GDM women, is a stronger predictor of their need for either instrumental delivery or caesarean section. Moreover, due to combined demographic, obstetric and medical data we have identified the local characteristics of women with GDM, which would help to predict their need for pharmacological therapy. This predictive model will improve streamlining of our patients’ care and improve utilization of local hospital resources. Further studies are still needed to identify the most effective and safe management of gestational diabetes within the public hospital setting.

## Data availability statement

The original contributions presented in the study are included in the article/supplementary material. Further inquiries can be directed to the corresponding author.

## Ethics statement

The studies involving human participants were reviewed and approved by The Southern Eastern Sydney Local Health District Human Research Ethics Committee (Study Reference No. RESP/15/107). Written informed consent for participation was not required for this study in accordance with the national legislation and the institutional requirements.

## Author contributions

Study design: MMB, AP, DB, AO'S. Study conduct: MMB. Data collection: MMB, AP. Data analysis: MMB, AP, DB, AKP. Data interpretation: MMB, AP, DB, AKP, AZ, AO'S. Drafting manuscript: MMB. Revising manuscript content: MMB, AP, DB, AKP, AZ, AO’S. Approving final version of manuscript: all authors. MB is the guarantor of this work. All authors contributed to the article and approved the submitted version.
